# The fall and rise of Group A *Streptococcus* diseases

**DOI:** 10.1017/S0950268818002285

**Published:** 2018-07-26

**Authors:** T. C. Barnett, A. C. Bowen, J. R. Carapetis

**Affiliations:** 1 Wesfarmers Centre for Vaccines and Infectious Diseases, Telethon Kids Institute, University of Western Australia, Perth, Australia; 2 Princess Margaret Hospital for Children, Perth, Western Australia, Australia; 3 Menzies School of Health Research, Charles Darwin University, Darwin, Northern Territory, Australia

**Keywords:** Necrotising facsciitis Group A *Streptococcus*, scarlet fever, streptococcal infections, *Streptococcus pyogenes*, toxic shock syndrome

## Abstract

*Streptococcus pyogenes* (or Group A *Streptococcus*, GAS) is a Gram-positive human pathogen responsible for a diverse array of superficial, invasive and immune-related diseases. GAS infections have historically been diseases of poverty and overcrowding, and remain a significant problem in the developing world and in disadvantaged populations within developed countries. With improved living conditions and access to antibiotics, the rates of GAS diseases in developed societies have gradually declined during the 20th century. However, genetic changes in circulating GAS strains and/or changes in host susceptibility to infection can lead to dramatic increases in the rates of specific diseases. No situations exemplify this more than the global upsurge of invasive GAS disease that originated in the 1980s and the regional increases in scarlet fever in north-east Asia and the UK. In each case, increased disease rates have been associated with the emergence of new GAS strains with increased disease-causing capability. Global surveillance for new GAS strains with increased virulence is important and determining why certain populations suddenly become susceptible to circulating strains remains a research priority. Here, we overview the changing epidemiology of GAS infections and the genetic alterations that accompany the emergence of GAS strains with increased capacity to cause disease.

## Introduction

*Streptococcus pyogenes*, also known as the Group A *Streptococcus* (GAS), is a remarkable human pathogen that causes a diverse spectrum of disease [1]. The majority of GAS diseases are relatively mild, superficial infections of the throat (pharyngitis, or ‘Strep throat’) and skin (impetigo, or ‘skin sores’). A rare, yet serious, consequence of these superficial infections occurs when the infecting strain gains access to deeper tissues, causing invasive infections that include the ‘flesh-eating disease’ necrotizing fasciitis and Streptococcal toxic shock syndrome (STSS), both of which have high case fatality rates. Another serious consequence of superficial GAS infections occurs when an abnormal immune response leads to post-streptococcal sequelae. These diseases include Acute Rheumatic Fever (ARF) and Acute Post-Streptococcal Glomerulonephritis, and can result in the long-term morbidities Rheumatic Heart Disease and chronic kidney disease that account for most of the global disease burden for this pathogen [2].

GAS strains are defined by the presence of the group A carbohydrate on their surface and are further classified based on serological or genetic differences in the surface M protein, encoded by the *emm* gene [1]. Over 220 different *emm* types have been documented and remarkable differences in *emm* type prevalence exist in different populations [3]. In particular, the dominant circulating *emm* types in industrialised populations are usually different and much less diverse than those that circulate in socio-economically disadvantaged populations (for review see [3]). Genetic differences within an individual *emm* type are also common, particularly as a result of differences in prophage (a bacteriophage genome inserted into the bacterial genome) gene content, or as a result of other horizontal gene transfer events [4].

Globally, GAS ranks in the top 10 infectious causes of human mortality, and a 2005 study attributed at least 517 000 patient deaths each year to this pathogen [2]. The majority of disease occurs in socio-economically disadvantaged populations in low and middle income (developing) countries, where overcrowding, poor nutrition and reduced living standards are thought to contribute to endemic GAS disease [2, 5]. Similar living conditions were prevalent in industrialised (developed) countries during the 19th and early 20th centuries when diseases such as scarlet fever and ARF were a considerable cause of morbidity and mortality [5–7]. In England in the mid-19th century, scarlet fever mortality at its peak was 174 per 100 000 across all age groups, with mortality in children less than 5 as high as 419 per 100 000 [8]. Scarlet fever was thus a greatly feared and fatal infectious disease, and children were admitted to hospitals in large numbers to manage symptoms and reduce transmission [9]. A similar high burden of ARF was seen in hospitals in industrialised contexts prior to the 1960s, after which rates began to decline to their current levels [10].

In industrialised countries, the burden of GAS disease in affluent populations has gradually decreased since the latter part of the 19th century as living conditions improved [5], but with persistence in disadvantaged populations within these countries [11, 12]. Improved sanitation, development of antibiotics and a reduction in poverty and household overcrowding have often been used to explain this decrease [5, 7, 9]. In recent years, however, a global resurgence of invasive infections and regional outbreaks of diseases such as scarlet fever have exacerbated public health concerns. In this review, we use the examples of invasive GAS infections and scarlet fever to highlight how genetic changes can lead to the emergence of new GAS strains with increased disease-causing potential. Much of what we know about these processes has come from a wealth of genome sequencing studies of isolates associated with disease emergence in industrialised populations. However, the precise host–pathogen interactions that contribute to each of these diseases are still being described and remain a research priority.

## Invasive GAS infections

Defined as infection of a normally sterile site, invasive GAS (iGAS) infections comprise a diverse array of severe diseases that include necrotising fasciitis, bacteraemia, STSS, pneumonia and cellulitis [1]. Beginning in the late 1980s, a marked increase in the number of iGAS infections was observed in the USA and Europe [13]. While this initial upsurge is believed to have been associated with the emergence of more virulent strains (e.g. M1T1), multiple *emm* types have been found to comprise iGAS infections in industrialised societies. A recent comprehensive study from the USA documented 99 unique *emm* types amongst 9557 iGAS infections; *emm* types 1, 12, 28, 89 and 3 were responsible for over half of these infections and *emm1* was the most prevalent causing approximately one in five infections [14]. A similar distribution of *emm* types associated with iGAS infections has been reported in other industrialised regions, including Europe [15] and Australia [16], while a broader range of *emm* types are associated with these infections in developing populations [3]. The *emm* types that dominate in industrialised countries are often not as prominent in low- and middle-income countries [3].

### Genetic changes associated with the emergence of iGAS strains

**Fig. 1.**
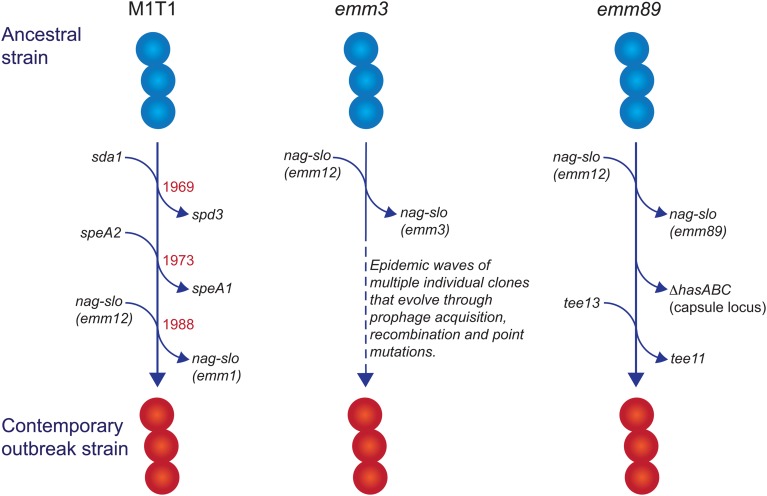
Evolutionary pathway of M1T1, *emm3* and *emm89* GAS strains with increased capacity to cause iGAS infections. For each example, multiple genetic events involving prophage acquisition, recombination and/or point mutations have resulted in contemporary strains with increased disease-causing capacity. Estimated dates for these events are provided for the M1T1 iGAS clone (data from [21]).

Recent genomic analyses of iGAS isolates from industrialised populations have identified conserved and *emm* type-specific features that likely have contributed to the emergence of these infections since the 1980s. These will be discussed below using the examples *emm1*, *emm3* and *emm89* strains (Fig. 1).

In industrialised settings, *emm1* strains are the most common *emm* type associated with iGAS infections in most epidemiological investigations [14–17]. Molecular typing [18] and whole genome sequencing [19–21] studies have revealed that a single clone (the so-called M1T1 clone) is responsible for almost all contemporary *emm1* infections since the global upsurge of disease in the 1980s. Compared with ancestral *emm1* strains (i.e. those isolated prior to this upsurge of disease), three separate genetic events define the M1T1 clone: acquisition of a prophage encoding a deoxyribonuclease (DNase) enzyme (Sda1, also called SdaD2) that has been shown to promote resistance to neutrophils; acquisition of the SpeA2 superantigen that has been proposed to aid pharyngeal colonisation; and a recombination event that replaces the chromosomal region encoding the secreted toxins NAD glycohydrolase (NAG) and Streptolysin O (SLO), which have been shown to mediate resistance to host polymorphonuclear cells [19, 20]. The recombination of the NAG-SLO region was recently identified as the final genetic event that led to the emergence of the M1T1 clone in the early 1980s [21]. As this region is conserved in other *emm* types commonly isolated from iGAS infections (e.g. 12, 89, 3), it has been proposed to constitute a key molecular event in the emergence of GAS strains with increased invasive disease capacity [21, 22].

### NAD glycohydrolase and Streptolysin O

SLO and NAG are a pair of co-transcribed secreted toxins that act synergistically to trigger cell death [23, 24]. SLO is a cholesterol-dependent cytolysin that inserts into the eukaryotic cell membrane, where it has been shown to facilitate translocation of NAG into the host cell [25]. Two variants of NAG exist in different strains [26, 27], a NADase-active variant that triggers cell death through depletion of NAD^+^, and a NADase-inactive variant that triggers cell death via programmed necrosis [24]. The recombination of the NAG-SLO region in the M1T1 clone resulted in three polymorphisms that increase the expression of the NAG and SLO toxins, and converted NAG to the NADase-active form [28]. For M1T1 GAS, these changes were shown to convey increased resistance to killing by human polymorphonuclear leukocytes and increased virulence in animal models of pharyngitis and necrotising fasciitis [28], suggesting that the NADase-active form contributes to increased GAS survival in sterile sites. Whether the NADase-active variant also directly influences disease severity in iGAS infections remains to be determined.

### *emm* type-specific changes

In addition to the replacement of the NAG-SLO gene region described above, some *emm* types frequently associated with iGAS infections have undergone additional genetic events that have contributed to their emergence. These changes will be discussed below using the examples of M1T1, *emm3* and *emm89* strains.

#### M1T1

In addition to its prevalence with iGAS infections, the M1T1 clone is also the most common strain associated with superficial pharyngeal disease in industrialised populations. This capacity, in part, has been attributed to the acquisition of the prophage-encoded SpeA2 allele. Whole-genome phylogenetic analysis suggested that acquisition of SpeA2 in the 1980s provided a powerful selection advantage that led to the global dissemination of the M1T1 clone [20]. SpeA2 differs from SpeA1 by a single amino acid change that results in significantly higher affinity for major histocompatibility complex class II HLA-DQ molecules [29]. While SpeA2 did not confer an increased ability to adhere to epithelial cells [20], SpeA1 has been shown to convey an increased ability of M18 GAS to colonise the nasopharynx of HLA-transgenic mice [30]. This phenotype was recently shown to be dependent on the presence of specific SpeA-responsive V*β*-specific T cells, suggesting that GAS uses SpeA (or superantigens in general) to manipulate V*β*-specific T cells in the nasopharyngeal environment to promote colonisation [31].

The transition of M1T1 GAS from superficial to invasive disease is facilitated by spontaneous mutations in the genes encoding the CovR/S two-component global regulatory system (reviewed in [32]). This ‘invasive switch’ results in abolished SpeB protease expression, along with concomitant strong transcriptional upregulation of multiple virulence-associated genes that are required for growth in sterile sites, including the *nag*, *slo* and *speA2* genes described above [33]. Also upregulated by CovR/S mutations are genes encoding M protein, capsule, a streptococcal inhibitor of complement (SIC), and interleukin-8 protease (SpyCEP/ScpC), all of which have documented roles in invasive disease pathogenesis [32]. In addition to the above, upregulation of the prophage-encoded DNase Sda1 was shown to be an important requirement for invasive disease and the accumulation of CovR/S mutations. Upregulation of Sda1 by CovR/S mutations allows M1T1 GAS to escape the effects of neutrophil-extracellular traps [34, 35] and Sda1 mutant strains lose the ability to acquire CovR/S mutations and switch to the invasive phenotype [36]. Thus, the three genetic events that comprise the evolution of the M1T1 GAS clone have led to global dissemination through improved pharyngeal colonisation (via SpeA2), a capacity to switch to a highly-virulent invasive phenotype (via Sda1) and an increased ability to survive in sterile sites (via Sda1, SLO and NAG).

#### emm3

Unlike M1T1, *emm3* GAS strains do not acquire CovR/S mutations as a consequence of an invasive switch [37], but do express high amounts of CovR/S-regulated proteins, including capsule, SLO, NAG and streptokinase, as a consequence of a mutant *rocA* allele, and this conveys increased survival within whole human blood [38]. Another contrast with the M1T1 clone is that *emm3* iGAS strains are not clonal and rapidly evolve into new clones that lead to epidemic waves of disease [39]. Numerous associated genetic changes have been described [39], including mutations in the M3 protein [40], the transcriptional regulators MtsR [41] and RopB [39] and loss or gain of prophages encoding superantigens [40]. Of note, the SpeA superantigen, in particular, has been associated with persistence of *emm3* clones, a role reminiscent of SpeA2 in the emergence of the M1T1 clone. Similarly, a 2008 upsurge in iGAS infections in the UK was found to involve a new *emm3* clone (ST15 lineage C) with a unique superantigen profile; lineage C strains lost the SSA superantigen and gained SpeC and the DNase Spd1 [42]. While these changes were not found to influence invasiveness in a mouse model of invasive disease, they did enhance nasopharyngeal infection and shedding [43], suggesting that the emergence of iGAS infections due to lineage C strains may primarily be associated with increased fitness and transmissibility.

#### emm89

GAS *emm89* strains have been increasingly associated with iGAS infections in the USA, Canada and Europe [22, 44–46]. This upsurge has corresponded with the emergence of a new genotypic variant (clade 3), characterised by two recent horizontal gene transfer events: replacement of NAG-SLO and loss of the *hasABC* gene region encoding the hyaluronic acid capsule [46], an unusual characteristic shared with *emm4* and *emm22* strains [47]. Clade 3 strains also belong to a different T serotype (T13; T serotype is based on the sequence of the major pilus subunit protein) than the ancestral strain (T11) [22]. The loss of the capsule biosynthesis genes is particularly intriguing, as capsule production has been shown to be essential for virulence of other GAS serotypes by virtue of its antiphagocytic properties (for review see [1]), and is required for the ability of M1T1 strains to switch to an invasive phenotype [48]. While capsule production appears to be dispensable for invasive infection by *emm89* strains when NGA and SLO are highly produced [28], the selective advantage conveyed by capsule loss in *emm89* strains is puzzling and remains to be determined. One possibility is that capsule loss may unmask other virulence determinants essential for iGAS infections by *emm89* strains.

## Scarlet fever

Scarlet fever is a disease that presents as a characteristic finely-papular erythematous rash, so-called ‘strawberry tongue’ and exudative pharyngitis [1]. While this disease is believed to be toxin-mediated, the toxin responsible for the characteristic rash has not been identified, although superantigens are believed to contribute (see below). Beginning in 2009 in Vietnam [49] and subsequently in South Korea, China and Hong Kong in 2011 [50–52], the rates of scarlet fever increased dramatically. Rates of disease increased approximately 10-fold in a single year in Hong Kong from 2010 to 2011 [50, 52] and have increased over 40-fold in South Korea from 2010 to 2015 [51]. Rates in mainland China increased approximately threefold in 2011 [50] and a similar increase was observed in the UK starting in 2014 [9]. While the reported magnitudes of disease in China and the UK were lower than in Hong Kong and South Korea, regional hotspots within these countries exhibited similarly high rates of disease [9, 53].

The reason for these regional outbreaks of scarlet fever remain unclear; whole genome sequencing of associated GAS strains from Hong Kong and the UK revealed the surprising finding that the outbreak strains were not clonal [50, 54]. While the majority of scarlet fever cases in Hong Kong and China were caused by *emm12* organisms [50], cases from the UK were associated with multiple emm types (*emm3*, *emm12*, *emm1* and *emm4* in order of decreasing prevalence [9]).

### Genetic determinants associated with scarlet fever outbreak strains

**Fig. 2.**
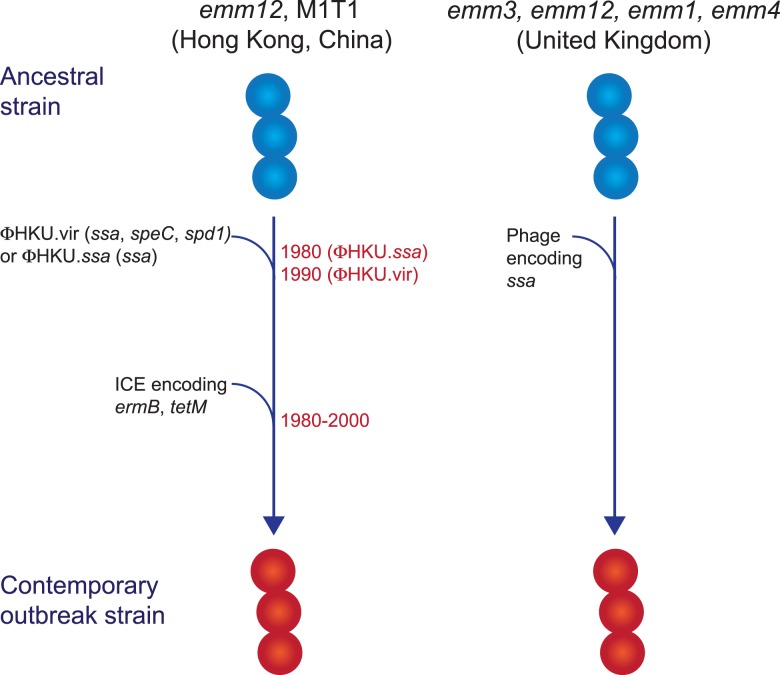
Evolutionary pathway of GAS strains with increased capacity to cause Scarlet Fever. Prophage acquisition of the superantigen SSA has resulted in contemporary strains with increased disease-causing capacity. Estimated dates for these events, which predate the scarlet fever outbreak by decades, are provided for the *emm12* scarlet fever strains from Hong Kong (data from [50]).

While multiclonal, what differentiated the scarlet fever outbreak strains from other circulating strains was the presence of novel prophages encoding the superantigens SSA and SpeC, and the DNase Spd1 (Fig. 2) [50, 52, 54, 55]. The *ssa* gene, in particular, was significantly associated with scarlet fever isolates from Hong Kong [50] and was present in all UK scarlet fever isolates [54]. Related prophages were found in multiple *emm* types [50, 54] and evidence suggests that transfer of the prophage ΦHKU.vir from *emm12* resulted in the emergence of M1T1 scarlet fever strains [55]. Likewise, a prophage similar to ΦHKU360.ssa was found in the majority of UK *emm12* scarlet fever isolates, as well as a single *emm28* isolate [54]. However, these prophages were not unique to scarlet fever strains and in China and Hong Kong had been circulating for decades prior to the scarlet fever outbreak [50]. Thus, the presence of *ssa* alone cannot account for the upsurge in scarlet fever. Nonetheless, the high incidence of this gene in scarlet fever isolates belonging to multiple *emm* types from two distinct geographical regions is remarkable, and determining the contribution of SSA to scarlet fever pathogenesis will be important for monitoring the emergence of this disease in other populations.

In addition to *ssa*, the majority of the scarlet fever outbreak strains from China and Hong Kong also harboured an integrative conjugative element encoding resistance to the antibiotics tetracycline, erythromycin and clindamycin [50, 52]. This element was not found in the UK scarlet fever isolates and likely reflects differences in prescribing characteristics in these regions. However, the presence of antibiotic resistance determinants in scarlet fever isolates from China and Hong Kong does raise the intriguing possibility that their selection by antibiotic use may have led to an increased prevalence prior to the onset of the outbreak in 2011, and potentially contributed to the magnitude of this outbreak.

Unfortunately, while considerable progress has been made in characterising the GAS strains responsible for the regional scarlet fever epidemics in Asia and the UK, the underlying trigger for these epidemics remains to be determined. The gene encoding SSA is the only genetic element common to these outbreaks, but is not unique to scarlet fever isolates and was circulating in GAS isolates long before these outbreaks started. It has long been known that only a proportion of a given population is susceptible to scarlet fever [56], and available evidence to date suggests that underlying co-infection and/or changes in host immune status might contribute to susceptibility to this disease. Uncovering this unknown factor remains a research priority in order to prevent the emergence of scarlet fever in other geographical regions.

## Summary/conclusions and unanswered questions

Despite the gradual decline of disease attributed to GAS in industrialised countries over the last several decades, the ongoing burden in developing countries and recent increases in individual disease manifestations in industrialised countries emphasise why GAS infections remain a significant global health problem. This review highlights how new GAS strains with increased fitness account for an upsurge of invasive GAS and scarlet fever infections in recent years. However, unique features associated with each of these diseases do not follow a single theme. Individual GAS clones are responsible for iGAS infections due to M1T1 and *emm89* strains, while *emm3* iGAS infections and scarlet fever are caused by multiclonal strains. Furthermore, the unknown role of non-GAS factors is highlighted by the emergence of scarlet fever outbreaks long after the expansion of the responsible GAS strains. In recent years, whole genome sequencing has provided an excellent description of the genetic changes that have accompanied disease increases, while also highlighting that these changes often differ between *emm* types. We are only beginning to understand the complex interplay of newly acquired genetic elements and altered expression of regulatory genes associated with disease. Whole genome sequencing remains an important tool for mapping emerging GAS strains and highlights genetic signatures that should be monitored. However, further experimentation is urgently needed to describe the pathogenic mechanisms of resurgent GAS infections and to reveal new strategies for the monitoring and treatment of these diseases. Finally, diseases that predominate in socio-economically disadvantaged populations remain very poorly understood. Diseases such as ARF and Acute Post-Streptococcal Glomerulonephritis remain a massive problem in socio-economically disadvantaged populations globally [2], yet the pathogenesis of these diseases is controversial (for reviews, see [1, 57–59]), and we still do not know how to identify the GAS strains that trigger these sequelae. As they are responsible for most of the GAS global disease burden, elucidating the pathogenic mechanisms of these diseases will be essential for the design of diagnostic tests and of safe and effective vaccines.
